# A Mouse Model of the Human Fragile X Syndrome I304N Mutation

**DOI:** 10.1371/journal.pgen.1000758

**Published:** 2009-12-11

**Authors:** Julie B. Zang, Elena D. Nosyreva, Corinne M. Spencer, Lenora J. Volk, Kiran Musunuru, Ru Zhong, Elizabeth F. Stone, Lisa A. Yuva-Paylor, Kimberly M. Huber, Richard Paylor, Jennifer C. Darnell, Robert B. Darnell

**Affiliations:** 1 Laboratory of Molecular Neuro-Oncology, The Rockefeller University, New York, New York, United States of America; 2 Department of Neuroscience, University of Texas Southwestern Medical Center, Dallas, Texas, United States of America; 3 Department of Molecular and Human Genetics, Baylor College of Medicine, Houston, Texas, United States of America; 4 Howard Hughes Medical Institute, The Rockefeller University, New York, New York, United States of America; The Jackson Laboratory, United States of America

## Abstract

The mental retardation, autistic features, and behavioral abnormalities characteristic of the Fragile X mental retardation syndrome result from the loss of function of the RNA–binding protein FMRP. The disease is usually caused by a triplet repeat expansion in the 5′UTR of the *FMR1* gene. This leads to loss of function through transcriptional gene silencing, pointing to a key function for FMRP, but precluding genetic identification of critical activities within the protein. Moreover, antisense transcripts (*FMR4*, *ASFMR1*) in the same locus have been reported to be silenced by the repeat expansion. Missense mutations offer one means of confirming a central role for FMRP in the disease, but to date, only a single such patient has been described. This patient harbors an isoleucine to asparagine mutation (I304N) in the second FMRP KH-type RNA–binding domain, however, this single case report was complicated because the patient harbored a superimposed familial liver disease. To address these issues, we have generated a new Fragile X Syndrome mouse model in which the endogenous *Fmr1* gene harbors the I304N mutation. These mice phenocopy the symptoms of Fragile X Syndrome in the existing *Fmr1*–null mouse, as assessed by testicular size, behavioral phenotyping, and electrophysiological assays of synaptic plasticity. I304N FMRP retains some functions, but has specifically lost RNA binding and polyribosome association; moreover, levels of the mutant protein are markedly reduced in the brain specifically at a time when synapses are forming postnatally. These data suggest that loss of FMRP function, particularly in KH2-mediated RNA binding and in synaptic plasticity, play critical roles in pathogenesis of the Fragile X Syndrome and establish a new model for studying the disorder.

## Author Summary

Missense mutations in human genes provide valuable insight into the genetic causes of disease. Fragile X Syndrome (FXS), a common genetic cause of autism and mental retardation, is usually caused by transcriptional silencing of the *FMR1* gene. The potential importance of single patient with a missense mutation (I304N) in an RNA–binding domain of the Fragile X protein, FMRP, has been questioned in part because he has a confounding liver disease. We introduced the I304N mutation into the endogenous *Fmr1* locus to create a mouse model of Fragile X Syndrome. We find that this mutation results in behavioral, electrophysiologic, and phenotypic features of the disease, loss of binding to RNA targets in the brain, and lower FMRP levels at a critical time during synapse formation. We conclude that loss of RNA binding and underexpression of FMRP are sufficient to cause the Fragile X Syndrome.

## Introduction

Missense mutations have been especially informative for establishing links between genetics and protein function in human disease. For example, missense mutations have advanced our understanding of the relationship between autism and mutations in genes including neuroligin-3 [1],[2], neurexin-1 [3], shank 3 [4], and MeCP2 [5]. Such mutations have not generally been of help in understanding the devastating effects of the loss of function of the Fragile X mental retardation protein (FMRP), which include complex behavioral deficits including mental retardation, autism, and seizures [6]. In nearly all cases, the Fragile X Syndrome is caused by transcriptional silencing of the fragile X mental retardation 1 (*FMR1*) gene as a result of CGG repeat expansion and hypermethylation of CpG islands in the 5′UTR region (reviewed in [7]), culminating in loss of FMRP expression. Moreover, antisense transcripts (*FMR4*, *AS-FMR1*) in the same locus have been reported to be silenced by the repeat expansion, raising the possibility that their loss of function may contribute to the syndrome [8],[9]. While this transcriptional silencing precludes structure-function analysis of FMRP, a single severely affected Fragile X Syndrome patient with a *de novo* missense mutation in FMRP has the potential to address this issue. This patient has marked macroorchidism, with testicular volume exceeding 100ml, and mental retardation, with IQ measured below 20, and harbors a mutation in a conserved isoleucine changing it to an asparagine (I304N) [10]. Nonetheless, uncertainty has surrounded the significance of this clinical observation, in part because only a single such patient has been described, and in part because this patient has a confounding liver disease [10].

Previous efforts at modeling defects in FMRP have centered on generation of an *Fmr1* null mouse (*Fmr1^tm1Cgr^*). This mouse has defects in synaptic plasticity [11]–[18] and long, thin dendritic spines [19],[20] similar to those found in human brain [21],[22]. Understanding the biochemical mechanism by which FMRP mediates proper synaptic plasticity and/or maturation is an area of intense interest.

Studies of FMRP have been necessarily restricted to *in vitro* and cell culture models, since the mouse model is a null. FMRP associates with polyribosomes in tissue culture cells [23]–[25] and mouse brain [26]–[28]. Moreover, FMRP, and the related protein FXR1P, associate with components of the RNA-induced silencing complex (RISC) in Drosophila and mammalian cells [29]–[32], and FXR1P is required to mediate miRNA-dependent translational activation in tissue culture cells [33],[34]. FMRP has also been proposed to have a role in mRNA transport, trafficking mRNA targets as granules from cytoplasm to synapses in a microtubule-dependent manner in primary neurons [35]–[37]. FMRP has also been suggested to regulate PSD-95 mRNA stability [38]. A common theme associated with these diverse cellular roles is that a critical function of FMRP is binding to specific RNA targets.

FMRP has functional domains involved in RNA binding, protein∶protein interactions and nuclear-cytoplasmic shuttling. FMRP RNA binding domains include two tandem KH-type domains (hnRNPK homology), an arginine and glycine-rich RNA binding domain (RGG box) [39],[40], and an N-terminal domain similar to Tudor/Agenet domains that may be involved in both RNA binding and protein-protein interactions [41]–[44]. Protein interaction domains include an N-terminal region responsible for homodimerization and heterodimerization with its autosomal homologs FXR1P and FXR2P [45],[46]. Finally, FMRP has a nuclear localization signal (NLS) mapped to approximately 100 nucleotides of the N-terminus [47], and a Rev-like nuclear export signal (NES) C-terminal to the KH domains, which, when mutated at critical leucines, causes accumulation of FMRP in the nucleus [48].

Interest in the RNA binding properties of the KH2 domain has been heightened by structural data suggesting that the human I304N mutation maps to the RNA binding pocket present in KH domains [49]. For example, the first structure of a KH domain (Nova KH3) bound to its RNA ligand demonstrated that the RNA binding pocket is supported by conserved hydrophobic amino acids, one of which corresponds to the isoleucine mutated in the I304N patient [50]. These observations have suggested that a key defect in FMRP loss-of-function is the loss of sequence-specific RNA binding, mediated through the FMRP KH2 domain [50],[51]. Here we address these issues by generating and analyzing a mouse (*Fmr1^tm1(I304N)Drnl^*, termed here *Fmr1^I304N^*) harboring the I304N mutation. We find that the I304N mutation phenocopies *Fmr1* null mice. The mutant protein has lost polyribosome association and RNA binding, and is present at reduced levels that vary with age, but are particularly low at P14, during synaptogenesis. These observations support the suggestion that sufficient levels of FMRP, and/or its RNA binding activity, are critical for normal cognition. Generation of the *Fmr1^I304N^* mouse provides a new model for understanding molecular defects in the disease, for screening potential therapies, and heightens interest in identifying FMRP-RNA interactions in the brain.

## Results

### Generation and characterization of *Fmr1* I304N mice

**Figure 1:**
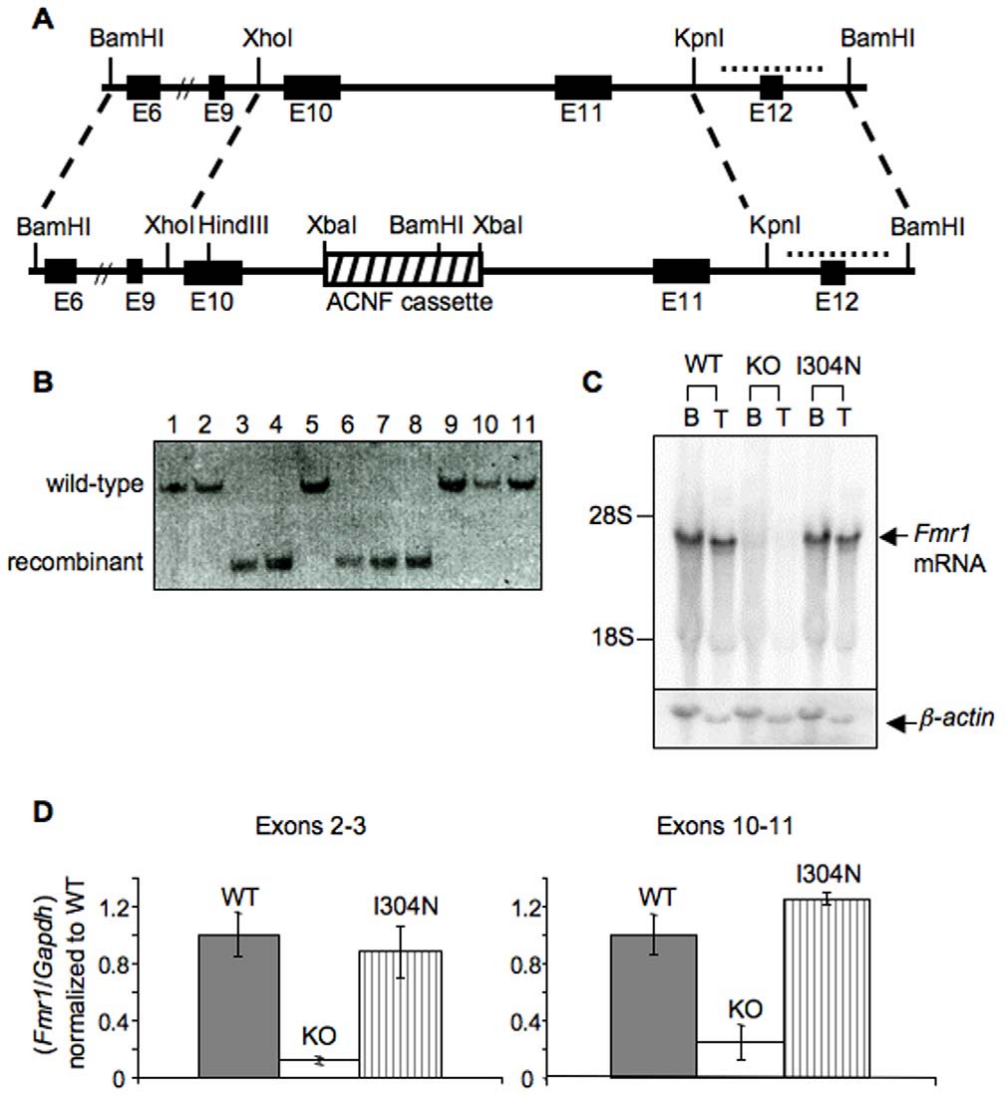
Generation of the *Fmr1* I304N knock-in mouse model. (A) The KH2-I304N *Fmr1* targeting construct introduces an isoleucine to asparagine mutation in exon 10. An Auto-Cre-Neo^R^ (ACNF) cassette for selection and auto-excision in the male germline was inserted in a non-conserved region of intron 10. (B) Southern blot analysis of representative ES cells transfected with the I304N *Fmr1* targeting construct. DNA from ES cell clones was digested with BamHI and probed for recombination by Southern blot using a probe outside the targeting construct (dotted line in (A)). The targeted allele, 2.7kb (clones 3, 4, 6–8), can be distinguished from the wild type locus, 9.6kb. (C) Brain (B) and testicular (T) mRNA from WT, *Fmr1* null (KO), and *Fmr1* ^I304N^ (I304N) mice was probed with an *in vitro* transcribed radiolabeled RNA probe anti-sense to the 3′UTR of *Fmr1* and visualized by Northern blot. β-actin mRNA was detected with an oligo probe. (D) RNA prepared from brain as in (C) was quantified by quantitative RT-PCR with two sets of primers encompassing different regions of the *Fmr1* mRNA, including primers spanning exons 2 to 3 (left panel) and primers spanning exons 10 to 11 (right panel), and normalized to *Gapdh* and WT mRNA levels. Error bars reflect the standard deviation from three mice of each genotype.

To generate *Fmr1^I304N^* mice we introduced the I304N mutation into the endogenous mouse *Fmr1* locus by homologous recombination (Figure 1A). An *Fmr1* KH2 I304N targeting construct, including a self-excising loxP-Auto-Cre-Neo^R^-loxP (ACNF) cassette which allows self-induced deletion of the selectable marker in the male germline [52], was electroporated into embryonic stem cells and 53 homologous recombinants were identified by Southern blot (Figure 1B). Germline chimeras were bred to generate *Fmr1^I304N^* mice. These were bred for greater than 10 generations into both FVB and C57BL/6J backgrounds.

We examined *Fmr1* mRNA expression in male FVB *Fmr1^I304N^* mice. Because the *Fmr1* gene is on the X chromosome these mice express only the I304N-mutant allele. Northern blot and quantitative RT-PCR analysis showed that I304N *Fmr1* mRNA was expressed at wild type levels and was of the expected size in both brain and testes (Figure 1C and 1D). Sequencing of RT-PCR products from the Fmr1^I304N^ mice confirmed the presence of the I304N mutation (data not shown).

*Fmr1^I304N^* mice had no overt phenotype, were fertile with normal litter sizes, and transmitted the mutant allele with the expected X-linked Mendelian segregation ratios. Histological examination of heart, spleen, liver, lung, kidney, adrenal glands, stomach, intestines, muscles, diaphragm, bladder, and thymus of *Fmr1^I304N^* mice revealed no macroscopic abnormalities nor microscopic lesions (data not shown). Further analysis focused on the brain and testes, as both organs are affected in Fragile X patients, and the I304N patient in particular. Histologic analysis of cerebellum, cortex, hippocampus and testes revealed no defects (Figure S1A, S1B, S1C, S1D, S1E, S1F, S1G, S1H, S1I, SIJ, S1K, S1L, S1M, S1N, S1O, S1P), similar to *Fmr1* null mice [53],[54].

**Figure 2:**
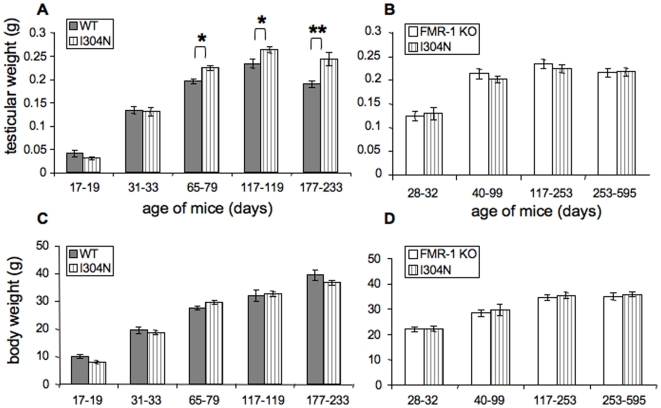
*Fmr1* ^I304N^ mice are macroorchid compared with WT littermates and have the same degree of macroorchidism as *Fmr1* ^tm1Cgr^ littermates. (A) Combined weights (W) of both testes of FVB.*Fmr1* ^I304N^ mice (n = 43) were compared with their wild type FVB littermates (n = 34). For statistical analysis, litters of similar ages were grouped together and data were subject to student's t-test. Age 17–19 days, weight (W) of the *Fmr1* ^I304N^ mice = 0.031±0.003g (n = 6), W(WT) = 0.042±0.007g (n = 3), p>0.05; age 31–33 days, W(*Fmr1* ^I304N^) = 0.130±0.009g (n = 6), W(WT) = 0.134±0.008g (n = 6), p>0.05; age 65–79 days, W(*Fmr1* ^I304N^) = 0.224±0.005g (n = 14), W(WT) = 0.195±0.005 (n = 13), *p<0.03; age 117–119 days, W(*Fmr1* ^I304N^) = 0.263±0.006g (n = 11), W(WT) = 0.234±0.010 (n = 6), *p<0.03; age 196–233 days, W(*Fmr1* ^I304N^) = 0.243±0.014g (n = 6), W(WT) = 0.190±0.007 (n = 6), **p<0.001. (B) Combined weights (W) of both testes of FVB.*Fmr1* ^I304N^ mice (n = 32) were compared with their FVB.*Fmr1* null littermates (n = 28). (C) Body weights from the same mice in (A). (D) Body weights from the same mice in (C).

Macroorchidism of greater than 25 ml combined testicular volume is present in more than 90% of adult males with Fragile X syndrome [55], and was particularly severe in the I304N patient whose testicular volume exceeded 100 ml [10]. We measured the testicular weights of adult *Fmr1^I304N^* mice compared to either WT or *Fmr1* null littermates. Macroorchidism was evident in *Fmr1^I304N^* mice compared to WT animals (12–28% increased weight), and the most pronounced differences were evident in older animals (Figure 2A). Testicular weight in *Fmr1^I304N^* mice was similar to, but surprisingly, no greater than that seen in FMR1 null mice (Figure 2B; [53],[54]). There was no significant difference in body weights between *Fmr1^I304N^* mice and wild type or *Fmr1* null littermates (Figure 2C and 2D). These observations indicate that the I304N mutation in mice phenocopies the macroorchidism evident in human patients, and suggests that the profound macroorchidism evident in the I304N patient may result from a combination of the I304N mutation and additional factors.

### Behavioral analysis of I304N mice

Modeling the human behavior deficits seen in Fragile X Syndrome in mouse models has proven challenging, although some marked differences between *Fmr1* null mice and normal littermates have been characterized [56],[57]. We compared *Fmr1^I304N^* and WT littermates in behavioral assays, blind to genotype. We assessed a battery of 11 behavioral tests that are well established measures of deficits in *Fmr1* null mice [58],[59]. These included measures of exploratory behavior, anxiety, acoustic startle and prepulse inhibition, conditioned fear, pain sensitivity, marble burying (as a measure of perseverative behavior), and susceptibility to audiogenic seizure. Results in the *Fmr1^I304N^* mice were consistent with lower levels of anxiety and greater repetitive/perseverative behavior (Table 1, Figure S2, and Text S1). Importantly, one of the most robust phenotypes in Fragile X null mice, increased susceptibility to audiogenic seizure, was evident in 18% of *Fmr1^I304N^* mice but not in WT mice; these results are comparable to or more severe than those reported in *Fmr1* null mice (see Text S1 for discussion). Taken together, our results indicate that in 10 of 11 tests, *Fmr1^I304N^* mice show similar responses to those reported for *Fmr1* null mice. Both are hyperactive, have increased perseverative behavior and audiogenic seizures, and reduced anxiety and startle reflexes relative to normal mice.

**Table 1:** Tabulation of behavioral test summary and comparison with historical findings on *Fmr1* ^tm1Cgr^ null mice.

Behavioral tests	I304N vs WT	KO vs WT
Activity in open field	↑	↑
Rearing in open field	-	-
Anxiety in open field	↓	↓
Anxiety in light/dark	↓	↓
Time to go to dark	-	-
Acoustic startle response	↓	↓
Pre-pulse inhibition	-	- (↑*)
Conditioned fear	-	-
Hotplate sensitivity to pain	↓	↓
Marble burying	↑	↑
Audiogenic seizure	18%	Reported*

Assays marked with an asterisk indicate areas of discrepancy with the literature. Prior studies of prepulse-inhibition [68] found a trend toward a decrease in PPI, as seen here, although others have reported it to be increased. The audiogenic seizure experiments were performed on 2–3 month old I304N mice and WT littermates, and the *Fmr1* null mice have not previously been reported to display audiogenic seizures beyond the age of 7 weeks in a C57Bl/6 background [89] although they consistently display audiogenic seizures at younger ages.

### I304N knock-in mice have altered synaptic plasticity in hippocampus

**Figure 3:**
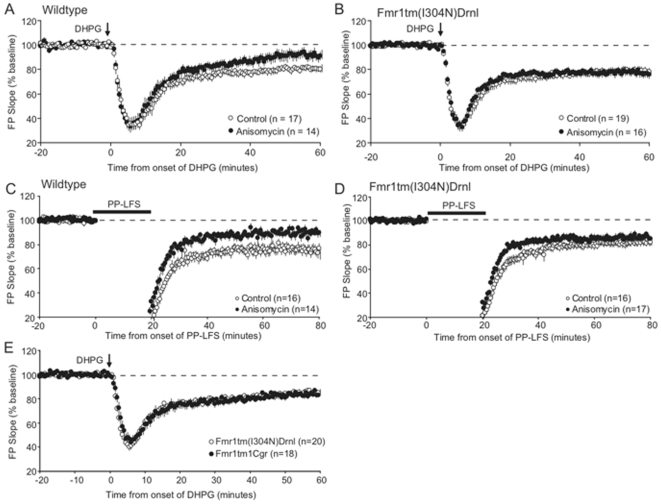
Protein synthesis-independent mGluR-LTD in *Fmr1* ^I304N^ mice. Evoked extracellular field potentials (FPs) from CA1 of acute slices from 30–90 day old wild-type and B6.*Fmr1* ^I304N^ littermates are plotted as a percent of baseline (pre-DHPG or PP-LFS). (A,B) Anisomycin inhibits DHPG-induced LTD in wild-type littermates, but has no effect in B6.*Fmr1* ^I304N^ mice (*; p = 0.02). (C,D) Anisomycin inhibits synaptically induced LTD (with PP-LFS; *; p = 0.004) in wild type mice, but not B6.*Fmr1* ^I304N^ mice. The magnitude of LTD between wild type and *Fmr1* ^I304N^ mice is not different under control conditions, but is enhanced in the presence of anisomycin (ANOVA and subsequent Fisher PLSD; p<0.05). (E) There is no difference in the degree of LTD elicited by DHPG stimulation between B6.*Fmr1* ^I304N^ mice and their *Fmr1* null littermates.

*Fmr1* null mice have defects in synaptic plasticity. The most well-studied relates to metabotropic glutamate receptor-dependent long term depression (mGluR-LTD) in hippocampal CA1 neurons that normally requires *de novo* protein synthesis in dendrites. In *Fmr1* null mice, mGluR-LTD elicited by either an mGluR agonist (DHPG) or electrical stimulation of Schaffer collateral inputs to CA1 neurons (paired pulse low frequency stimulation (PP-LFS)) is enhanced [11], and no longer requires protein synthesis [60],[61]. We assayed the protein synthesis requirement for both chemically and synaptically induced mGluR-LTD in acute hippocampal slices prepared from *Fmr1* ^I304N^ mice and their wild type littermates. Pre-incubation with the protein synthesis inhibitor anisomycin inhibited both DHPG (p = 0.003) and PP-LFS (p = 0.003) induced LTD in wild type mice (Figure 3A and 3C), but had no effect on the establishment of LTD in *Fmr1* ^I304N^ mice (Figure 3B and 3D). LTD magnitude in the absence of anisomycin was not different between *Fmr1* ^I304N^ and wild type littermates. We also found no significant difference between wild type and *Fmr1* null mice in these studies, consistent with the lack of enhanced LTD in the I304N mice (see Discussion).

Finally, since the I304N patient is more severely affected than typical Fragile X patients, we examined whether mGluR-LTD is enhanced in the *Fmr1* ^I304N^ relative to *Fmr1* null mice. We compared DHPG-elicited LTD measurements in *Fmr1* ^I304N^ and *Fmr1* null littermates, and found that there was no difference in the degree of LTD elicited (Figure 3E).

Taken together, given the degree of similarity in the phenotypes between *Fmr1* ^I304N^ and *Fmr1* null mice, including their behavior, macroorchidism and altered synaptic plasticity, we conclude that the I304N mutation in FMRP is sufficient to cause symptoms of the Fragile X Syndrome. We next used this mouse model to address the mechanism by which this missense mutation in the KH2 RNA-binding domain of FMRP results in the Fragile X phenotype.

### I304N FMRP levels in the I304N mouse

**Figure 4:**
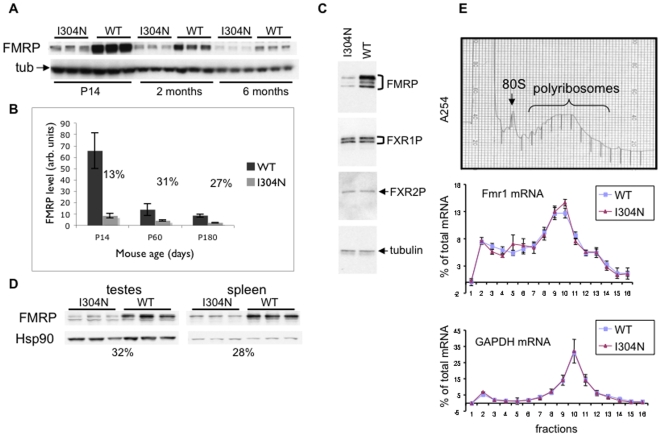
Expression of FMRP in *Fmr1* ^I304N^ and WT littermates. (A) 50 ug of brain lysate from three *Fmr1* ^I304N^ mice and three wild type littermates at P14, 2 mo, and 6 mo of age, were analyzed by Western blot for steady state FMRP expression using the anti-FMRP C-terminus antisera ab17722 (Abcam) and gamma-tubulin as a loading control. (B) Signal was quantified by chemiluminescence using Versadoc imaging and the percentage of I304N-FMRP signal compared to wild type FMRP is indicated. Error bars reflect the standard deviation of three mice. This experiment has been repeated several times and with different antibodies to FMRP (Chemicon ab2160 (1C3), 2F5 [84], and 7G1-1 [85] with consistent results. (C) 50 ug of brain lysate from P14 mice of the indicated genotype (littermates) was analyzed for expression of FMRP with ab17722, FXR1P, FXR2P, and gamma-tubulin. (D) 50ug of testes or spleen lysate from the same animals at P60 was analyzed in the same way with ab17722, and normalized to Hsp90 levels. (E) Brain lysates for polyribosome analysis were prepared from *Fmr1* ^I304N^ mice and WT littermates, and fractionated over linear 20%–50% sucrose gradients. The levels of *Fmr1* mRNA and *Gapdh* mRNA were quantified in each fraction by quantitative RT–PCR using the ΔΔCt method. An A254 absorption profile from one of the WT mice is shown in the upper panel and the 80S monosome and polyribosomes are indicated. Other gradients were indistinguishable within a WT and I304N littermate pair. Relative mRNA level in each fraction was plotted as a percentage of total mRNA to illustrate its distribution over the polyribosome gradient. Error bars reflect three technical replicates from a single littermate pair. The experiment has been repeated with additional littermate pairs, but cannot be plotted on one graph due to variable fraction collection between experiments. Representative graphs are shown.

We examined I304N-FMRP expression in the brain, testes, and spleen by Western blot analysis. At 2 months of age, I304N-FMRP was expressed at ∼30% of normal levels in brain and remained at ∼30% of WT levels at 6 months of age (Figure 4A and 4B). In younger mice (P14), WT FMRP levels were much higher, while I304N-FMRP was expressed at levels only slightly higher than in older mice, leading to a relatively larger difference between WT and I304N FMRP levels in the second postnatal week (∼13% of the WT level; Figure 4A and 4B). We found no evidence for a compensatory increase of the FMRP homologues, FXR1P and FXR2P, in I304N mice (Figure 4C). I304N-FMRP was also present at lower steady-state levels than the WT protein in other tissues (∼30% in testes and spleen at 6 months of age; Figure 4D).

Decreased protein steady-state levels with normal mRNA levels (Figure 1C and 1D) suggests that I304N-FMRP may either be synthesized more slowly or turned over more rapidly. We analyzed whether I304N *Fmr1* mRNA was being translated by comparing its distribution on polyribosomes with WT *Fmr1* mRNA in P14 mice. Quantitative RT-PCR analysis of mRNA levels showed that I304N *Fmr1* and WT *Fmr1* mRNA had similar distributions across 16 sucrose gradient fractions (Figure 4E). These observations suggest that the lower I304N-FMRP levels do not relate to translational control, but may relate to increased turnover of the mutant protein, particularly in younger mice.

### I304N FMRP function in the I304N mouse

**Figure 5:**
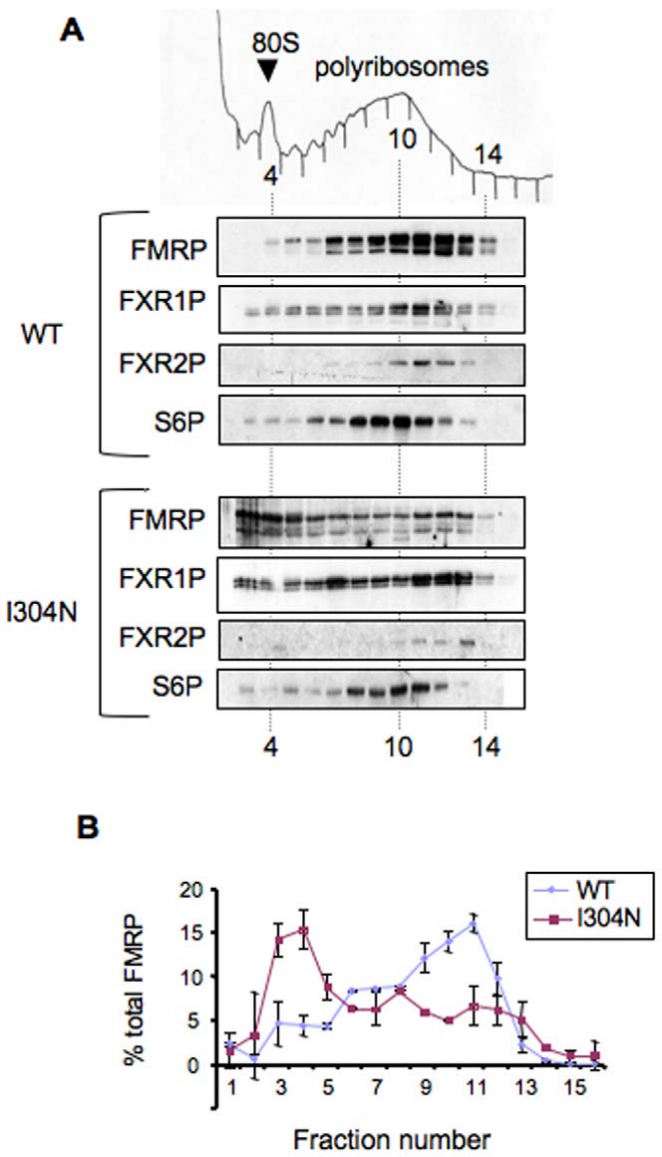
Endogenous I304N-FMRP is dissociated from polyribosomes in mouse brain. (A) Mouse brain cytoplasmic extracts from WT and *Fmr1* ^I304N^ littermates (second postnatal week) were separated on 20–50% linear sucrose gradients. The positions of the 80S monosome and polyribosomes are indicated on the A254 profile from each gradient (top panel). FMRP (detected with either 17722 or 7G1-1 antibody, to insure no crossreactivity with FXR1P and FXR2P)), FXR1P (ML13 antibody), and FXR2P (1G2 antibody) distributions were analyzed by Western blot. Ribosomal protein S6 (S6P) distribution confirms the integrity of the polyribosomes. (B) Quantification of chemiluminescence by Versadoc imaging of the Western blot of FMRP distribution from WT and *Fmr1* ^I304N^ littermates; values reflect the percentage of FMRP present in each fraction relative to total FMRP in the gradient. Errors bars reflect the standard deviation of the technical replicates. Quantification of this data revealed that very little wild type FMRP was present in fractions containing less than 2 ribosomes per transcript (fractions 1–5) and more than 55% was present on heavy polyribosomes (fractions 9–13), while 44% of total I304N-FMRP was in the corresponding light fractions (1–5) with a corresponding loss from the heavy polyribosomes. The experiment was repeated multiple times with additional littermate pairs with very similar results.

FMRP associates with polyribosomes in tissue culture cells [23]–[25] and in brain [26]–[28], suggesting that the protein may regulate mRNA translation. In contrast, in lymphoblastoid cell lines derived from the I304N patient, and in cells transfected with an EGFP-tagged I304N-FMRP reporter construct, mutant FMRP no longer associates with polyribosomes [25],[62]. To examine whether the I304N mutation affected endogenous FMRP-polyribosome association in the brain, we examined the distribution of the mutant protein in the brains of *Fmr1* ^I304N^ mice. A_254_ traces of polyribosomes separated by sucrose density centrifugation revealed no difference between wild type and *Fmr1* ^I304N^ mouse brain, suggesting that global translation status was normal in the mutant mice (data not shown). However, I304N-FMRP was largely dissociated from polyribosomes in mouse brain (Figure 5A), and there was a reciprocal increase in I304N-FMRP present in lighter polysome fractions (Figure 5B). We also found that the I304N mutation in FMRP does not significantly affect FXR1P or FXR2P polyribosome association in mouse brain (Figure 5A), suggesting that their polysome-association is not FMRP-dependent. These results are consistent with previous findings in patient lymphoblastoid cell lines [25], and suggest that the I304N mutation impacts the normal function of FMRP on polyribosomes.

### The endogenous I304N-FMRP complex is abnormally small due to loss of RNA binding by mutant FMRP

**Figure 6:**
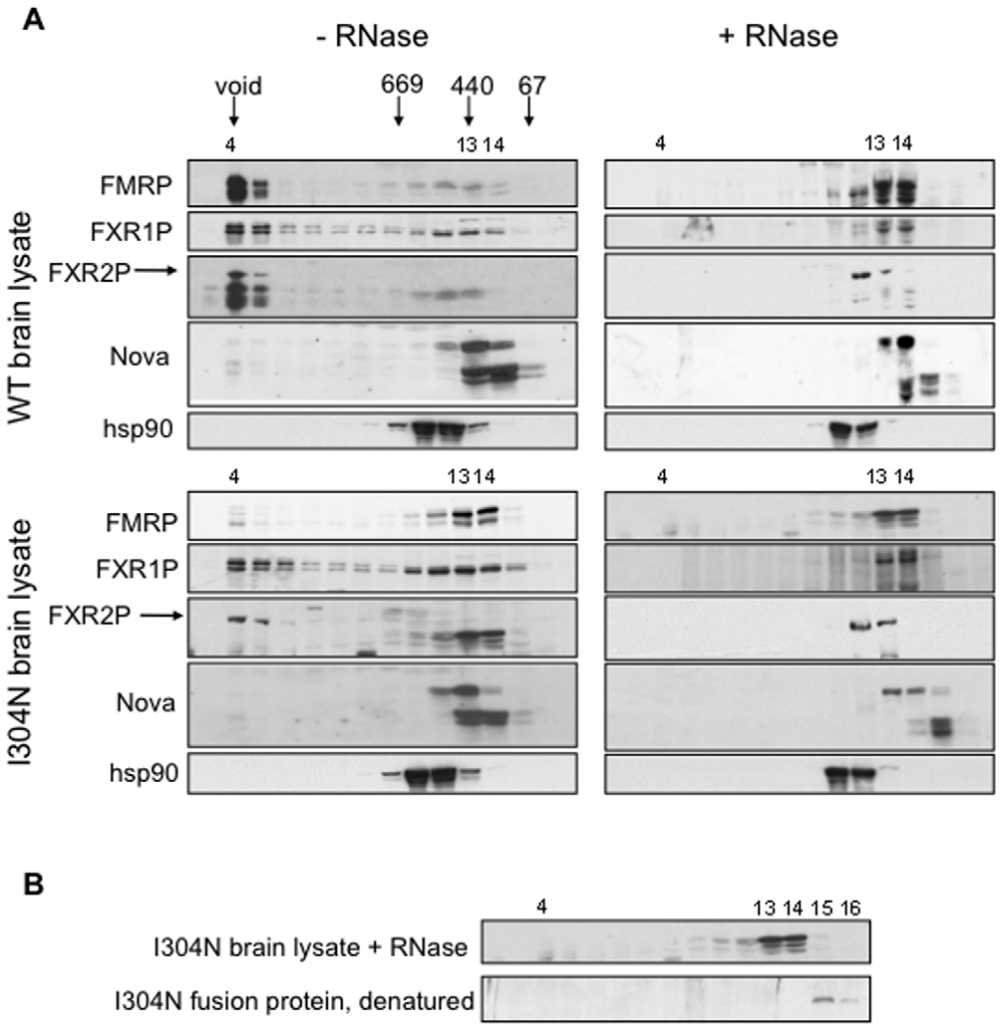
Endogenous I304N-FMRP is in an abnormally small and RNase resistant complex. (A) The migration of standards including blue dextran for the void volume, and proteins of 669, 440, and 67 kDa used to calibrate the Superose 6 3.2/30 column are shown. Brain lysates were prepared from WT and *Fmr1* ^I304N^ littermates (P22), treated with EDTA to disrupt polyribosomes, and treated with or without an overdigestion with RNases as indicated, and applied to a Superose 6 column sequentially. Two gradients for each condition were pooled to obtain enough material from eluted fractions for Western blot. Column fractions were TCA precipitated and Western blotted for wild type or I304N-FMRP (1C3), FXR1P (ML13), FXR2P (1G2), Nova (human patient antisera) and hsp90. (Upper left) The wild-type FMRP mRNP complex eluted in the void volume of the column (fraction 4) corresponding to a size of >40,000 kDa, as do most of FXR1P and FXR2P. FXR2P is shown by the upper band (arrow) as the FMRP blot was reprobed with 1G2 without stripping. (Lower left) I304N-FMRP is in a much smaller complex of 100–400 kDa (fractions 13 and 14). Most of FXR1P in the *Fmr1* ^I304N^ lysate remains in the void volume though there is a small increase in fractions 11–14. FXR2P (arrow) remains in the void volume. (Upper right) After complete RNase A and T1 digest, the wild type FMRP RNase-resistant complex migrates at the size of the native I304N FMRP complex (fractions 13 and 14) as does FXR1P. The RNase-resistant FXR2P complex migrates in fractions 12 and 13. (Lower right) The I304N-FMRP complex does not shift in apparent size after RNase treatment. FXR1P and FXR2P shift in a similar way in the *Fmr1* ^I304N^ lysate as they do in the presence of wild-type FMRP. In all conditions, hsp90 migrates in fractions 11 and 12. An irrelevant RNA binding protein, Nova, runs as several isoforms, two of about 68 kDa and three of approx. 52 kDa. Their migration is identical in *Fmr1* ^I304N^ and wt lysates (fraction 12–14), and they are shifted to smaller size after RNase treatment in both lysates (fraction 13–15). (B) The RNase-resistant complex containing I304N-FMRP is larger than a monomer of I304N-FMRP alone. I304N fusion protein was denatured, added to a mouse brain lysate, and run on the same system to determine the migration of a monomer of I304N-FMRP. It was detected in fraction 15–16 using an anti-HisTag antibody.

It has been suggested that the I304N mutation renders FMRP incapable of forming normal mRNP complexes in cultured cells [25]. We analyzed endogenous WT or I304N-FMRP particle size by Superose 6 gel filtration of mouse brain cytoplasmic extracts prepared in EDTA to release ribosomal subunits from mRNA. Wild type FMRP was found in the void volume of the Superose 6 column, indicating that FMRP is normally present in a complex of greater than 40,000 kDa (Figure 6A, upper left panels). In contrast, the majority of I304N-FMRP was shifted into a smaller complex eluting at approximately 100–300 kDa (Figure 6A, lower left panels). This complex may correspond to a small (<440kD) I304N complex observed in I304N patient-derived lymphoblastoid cells [25]. Mutant I304N-FMRP had no significant effect on the apparent size of FXR1P or FXR2P complexes (Figure 6A, left panels).

To assess whether the loss of I304N-FMRP from larger complexes might result from a loss of protein-RNA interaction, we treated brain cytoplasmic extracts with excess RNase prior to Superose 6 gel filtration. Under these conditions, the WT FMRP complex size was reduced to the size of the I304N-FMRP complex (Figure 6A, upper panels, compare fractions 4–5 and 13–14), but the I304N complex did not change in apparent size (Figure 6A, lower panels); similar results were seen when EDTA was omitted from the lysis buffer (data not shown). This suggests that relative to FMRP, the I304N-FMRP in mouse brain has lost most or all of its ability to associate with RNA. We cannot rule out binding to small RNAs that would not affect migration on Superose 6 columns (those less than ∼200 nucleotides (66 kDa), which we estimate would shift Superose 6 migration). We note that a small amount of I304N-FMRP is present in the void volume in an RNase sensitive manner, which could be due to heterodimerization with other RNA binding proteins or residual RNA interactions from other FMRP RNA binding domains.

### Mouse brain I304N FMRP retains some functional domains

To assess whether the I304N protein retains reported biologic activities *in vivo*, we evaluated whether it was competent to interact with protein partners. We first compared the size of the RNase-treated I304N FMRP complex with that of denatured, recombinant I304N-FMRP added to mouse brain extract by Superose 6 gel filtration. Endogenous mouse brain I304N-FMRP was found in a complex significantly larger (fractions 13 and 14) than the I304N recombinant protein added to the same extract (fraction 15), suggesting that the native I304N protein in brain is capable of protein interactions independent of RNA binding (Figure 6B).

**Figure 7:**
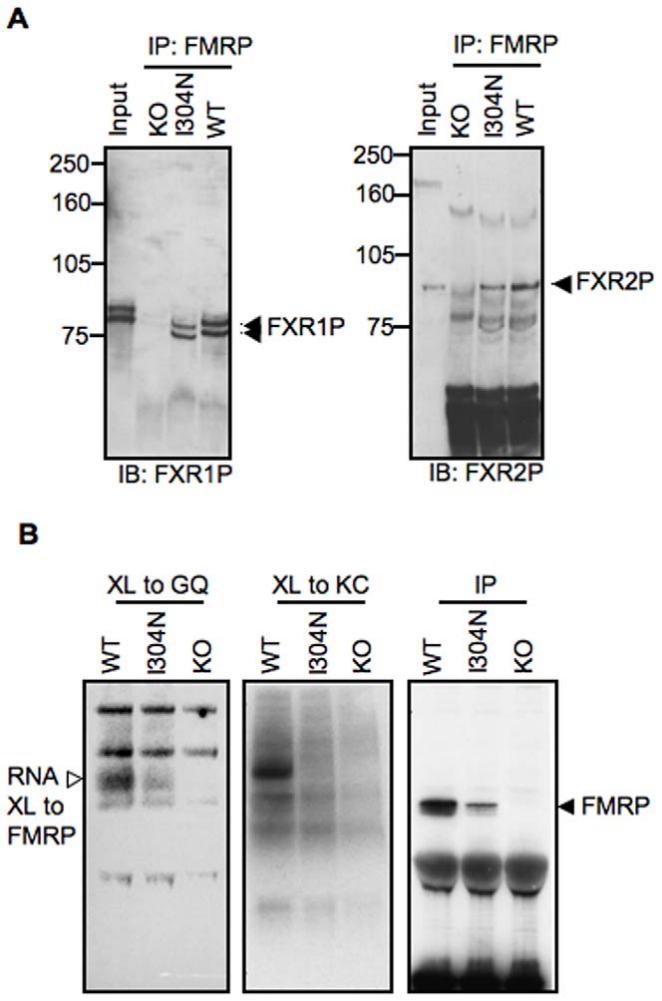
I304N-FMRP retains the ability to heterodimerize and bind G-quartet RNA. (A) Brain lysates for IP were prepared from WT, *Fmr1* ^I304N^ (I304N) and *Fmr1* null (KO) mice, and FMRP IPed with 7G1-1 antibody. Immunoprecipitates were probed by Western blot for co-IP of FXR1P (left panel) and FXR2P (right panel). Approximately 5% of the input lysate was run in lane 1. (B) Brain extracts from adult WT, *Fmr1* ^I304N^ (I304N), and *Fmr1* null (KO) mice were incubated with radiolabeled RNA (G-quartet RNA (left panel) and kissing complex RNA (middle panel)) generated by *in vitro* transcription in the presence of ^32^P-UTP. Samples were UV crosslinked (XL), immunoprecipitated with FMRP specific 7G1-1 antibody, run on SDS-PAGE gels, transferred to nitrocellulose and exposed by phosphorimaging to detect radiolabeled RNA crosslinked to FMRP (open arrowhead). The same immunoprecipitates (IP) were probed for FMRP by Western blot using antibody 2F5 (right panel, filled arrowhead).

FMRP was previously found to heterodimerize with its two autosomal homologs, FXR1P and FXR2P, by yeast two-hybrid assays [46]. *In vitro* studies indicated that I304N FMRP retains the ability to heterodimerize with FXR1P and FXR2P [63]. We analyzed the ability of endogenous I304N-FMRP to heterodimerize in mouse brain by immunoprecipitating I304N protein and assaying for co-precipitating FXR1P and FXR2P by Western blot. These experiments demonstrated that FXR1P and FXR2P co-precipitated with WT and I304N-FMRP, but not in control IPs from FMRP null brains (Figure 7A). Less FXR1P and FXR2P are co-precipitated by I304N-FMRP as compared with wild-type FMRP, but this is likely to be accounted for by the lower FMRP levels in the I304N mutant mouse (Figure 4, Figure 7B, third panel).

*In vitro* RNA selection studies identified a kissing complex RNA that is bound with high affinity by the FMRP KH2 domain [51] and a G-quartet RNA ligand for the C-terminal RGG–type RNA binding domain [64],[65]. Recombinant I304N-FMRP produced in insect cells has been shown to bind to G-quartet RNA, but not the kissing complex RNA [51]. We therefore examined endogenous I304N-FMRP in mouse brain to determine whether it recapitulated these RNA binding properties. Radiolabeled G-quartet or kissing complex RNA synthesized by *in vitro* transcription was added to mouse brain lysates, UV-crosslinked, immunoprecipitated with an antibody against FMRP and crosslinked RNA detected by autoradiography after SDS-PAGE. A radiolabeled FMRP∶RNA complex was seen specifically in the immunoprecipitate of the I304N-FMRP extract crosslinked to the G-quartet RNA, but little or no I304N protein was crosslinked to kissing complex RNA (Figure 7B). When compared with the wild type FMRP, the reduced radioactive signal from G-quartet RNA crosslinked to the I304N-FMRP in mouse brain was consistent with lower I304N-FMRP levels in the knock-in mice (Figure 7B, third panel). Point mutants of G-quartet or mutant kissing complex RNAs, which are not bound by recombinant FMRP *in vitro* [51],[64] did not crosslink to either endogenous wild type or I304N-FMRP in mouse brain (data not shown). Taken together, these data indicate that I304N-FMRP in mouse brain retains some normal properties, as it is competent to bind both protein and, via its RGG-domain, to bind G-quartet RNA. RGG-domain RNA binding to RNA over 200nt is not evident in the I304N mouse, suggesting either that it plays a minor or dependent role to KH2 binding to large RNAs. Therefore a major biochemical defect in the *Fmr1* ^I304N^ mouse is the loss of KH2-dependent RNA binding. While other interpretations cannot be ruled out, including the loss of KH2-dependent interaction with a protein partner, taken together our data suggest that the resulting loss of polysome association and, presumably, proper regulation of translation of FMRP mRNA targets, is most likely to contribute to the phenotype of the *Fmr1* ^I304N^ mouse.

## Discussion

The Fragile X Syndrome is usually caused by a triplet repeat expansion in the 5′UTR of the *FMR1* gene leading to transcriptional silencing of the *FMR1* mRNA and failure to produce the FMRP protein product. A severely affected patient has offered possible insight into a key function of FMRP, as he harbors a missense (I304N) mutation in the KH2 RNA binding domain [10]. The I304N mutation has previously been shown to abrogate RNA binding of similar KH-type RNA binding domains *in vitro*, suggesting that the disease symptoms in this patient are caused by the loss of FMRP KH2 domain sequence-specific RNA binding. However, it has not been clear whether the I304N mutation abolishes RNA binding in neurons nor that this mutation alone causes the Fragile X symptoms in this individual patient, as his clinical picture is complicated by an unrelated familial liver disease, X-linked liver glycogenosis due to phosphorylase kinase deficiency. To address these issues, we have generated and analyzed a mouse model harboring the I304N mutation in the endogenous *Fmr1* locus. These mice exhibit many of the phenotypic (macroorchidism), electrophysiologic and behavioral changes of *Fmr1* null mice, and thereby indicate that the I304N mutation is sufficient to phenocopy transcriptional silencing of the *Fmr1* gene. Surprisingly, we find both that FMRP RNA binding is lost in the brains of these mice, establishing a connection between KH2 RNA binding and the neurologic disorder, and that protein levels are markedly reduced at P14, a time of synaptogenesis in the neocortex and other brain areas.

### I304N mice have a *Fmr1* null-like phenotype

Behavioral changes in the *Fmr1* null mouse relative to either wild-type or mutant littermates have been well described. We compared the *Fmr1* ^I304N^ mouse to wild-type littermates on the same background and using many of the same assays employed for extensive behavioral testing of the *Fmr1* null mouse, the *Fxr2* null mouse, double knockout of both *Fmr1* and *Fxr2*, and a mouse overexpressing human FMRP from a transgenic YAC construct [53], [66]–[69]. In most cases the phenotype of the KH2 mutant *Fmr1* ^I304N^ mice was similar to the previously published phenotype of the *Fmr1* null mice, including increased audiogenic seizure rates, decreased acoustic startle responses, and assays indicating greater exploratory behavior, decreased anxiety responses and increased perseveration. Our results measuring PPI, while consistent with *Fmr1* null mice assessed in our laboratory [68], differed from those of some investigators [70],[71]; it is possible that strain differences may account for some of this discrepancy. In summary, our behavioral assays of *Fmr1* ^I304N^ mice indicate that they show abnormalities in the same tests, in the same direction, and to similar levels in all assays previously performed in our laboratory (Table 1), strongly supporting the conclusion that the I304N mutation is sufficient to phenocopy loss of the *Fmr1* gene.

Metabotropic glutamate receptor-dependent LTD is a hippocampal synaptic plasticity paradigm that relies on rapid protein synthesis in dendrites [72]. Previous work in the *Fmr1* null mouse demonstrated that LTD is enhanced and independent of new protein synthesis and translational regulators, such as ERK and Homer [11],[13],[16],[60],[61],[73]. From these studies, it was suggested that FMRP regulates the translation of the dendritic mRNAs required for mGluR-LTD expression. Here we demonstrate that LTD induced with either chemical or synaptic stimulation is independent of protein synthesis in the *Fmr1* ^I304N^ mice, recapitulating the *Fmr1* null phenotype (Figure 3). This suggests that FMRP must interact with polyribosomes or kissing-complex RNAs for normal mGluR-LTD regulation. Alternatively, the effects on LTD could be due to a hypomorphic expression of I304N-FMRP. It is unclear why we did not detect alterations in the magnitude of LTD between *Fmr1* ^I304N^ mice and wildtype littermates or *Fmr1* null littermates as demonstrated previously [13],[16],[60],[61]. We also found no significant enhancement of LTD between wild type and *Fmr1* null littermates in the current study. This may be due to the fact that older mice were used in this study (40–90 day) than in previous work (21–35 day) or other factors such as stress levels, which are known to impact mGluR-LTD magnitude [74]. Subtle genetic background differences may also play a role. Notably, the independence of LTD on protein synthesis, as seen here in the *Fmr1* ^I304N^ mice appears to be a more robust and reproducible phenotype in *Fmr1* null mice in comparison to enhanced LTD magnitude [73],[75].

Macroorchidism is a profound clinical finding in postpubertal Fragile X patients, affecting more than 90% of adult male patients [55]. In mouse models, the Fragile X null mouse has a 20–25% increase in testicular size [53],[54],[76] which is rescued by a wild type human FMRP transgene [53]. The *Fmr1* ^I304N^ mice display the same degree of macroorchidism as their null counterparts, and this increases with age, as in the *Fmr1* null mice [54] and in the human patients [6], supporting the conclusion that the *Fmr1* ^I304N^ mutation is sufficient to phenocopy the Fragile X Syndrome.

### Biochemical analysis of I304N mice

Steady-state levels of endogenous I304N-FMRP were found to be decreased relative to WT FMRP. While all of the characteristic isoforms of FMRP are observed in the *Fmr1* ^I304N^ tissues, they are expressed at lower levels than in wild type littermates. The post-transcriptional reduction in steady state levels of I304N-FMRP compared with mRNA levels has also been observed in two lines of I304N-FMRP BAC transgenic mice (data not shown), and in I307N-*dfmr1* flies, which have the analogous mutation to I304N-FMRP in mammals [77]. Taken together, these data suggest that decreased protein levels are intrinsic to the mutation rather than a result of our genetic manipulation.

Lower steady state levels of I304N FMRP in brain and testes are surprising in light of previous data demonstrating that I304N FMRP is expressed at normal levels in EBV transformed lymphoblastoid cells from the patient with the I304N mutation [25] and may be due to the fact that a different cell type was studied or that EBV transformation altered normal FMRP expression. We find that steady state levels of I304N-FMRP are too low in cultured primary neurons to permit standard pulse-chase immunoprecipitation experiments to quantify I304N FMRP synthesis and turnover (data not shown). Another means of assessing FMRP synthesis is to analyze the distribution of its mRNA on polyribosome sucrose gradients. We have shown that I304N-*Fmr1* mRNA has a normal profile on polyribosomes compared with wild-type littermates (Figure 4E), suggesting that decreased protein synthesis is not likely to account for decreased protein levels. It has been reported that FMRP can bind a G-quartet motif in the coding sequence of its own mRNA, inhibiting its translation [65]. However, we find no evidence to support the consequent prediction that in the I304N mouse there would be an increase in translation of the I304N-*Fmr1* mRNA. Taken together, it seems most likely that the observed decrease in I304N protein levels is due to increased turnover of the mutant protein.

Interestingly, the decrease in I304N FMRP levels is much more pronounced in mice at P14, relative to older mice (Figure 4A and 4B). This correlates with the observation that there are transient alterations in the morphology of dendritic spines in *Fmr1* null mice [19]. This suggests that the biochemical defect present in I304N FMRP may be compounded by a decrease in its levels during synaptogenesis, and that the phenotype may result from a combination of these effects.

### I304N FMRP is defective in polyribosome association and RNA binding

Several attempts have been made to assess the effect of the I304N mutation on FMRP RNA binding and function. Recombinant or *in vitro* translated I304N FMRPs show significantly decreased binding to ribohomopolymers or *in vitro*-selected RNA ligands [40],[51],[78]. Other studies have found that recombinant I304N FMRP produced in insect cells retained some ribohomopolymer binding, but with decreased binding to poly-U [79]. The I304N mutation in FMRP abrogates binding to high affinity *in vitro* selected KH2 target RNA ligands (kcRNA) but not RGG target (G-quartet) RNAs, as assessed with both full-length FMRP and isolated RNA binding domains [51],[64], indicating that KH-specific RNA interactions are lost in the I304N mutant *in vitro*. However, in lymphoblastoid cell lines derived from I304N patients, some I304N-FMRP was able to be captured on oligo-dT columns, which was interpreted as showing that I304N mRNA association was intact [25]. We also find that some I304N FMRP is retained in the void volume of the Superose 6 column in an RNase-dependent manner (Figure 6), which may be due to heterodimerization with FXR1/2P (Figure 7A; [63]), or to residual RNA binding from the RGG domain (Figure 7B).

The conclusion that I304N FMRP KH2 domain fails to bind RNA *in vivo* is consistent with structural studies of several RNA-binding proteins suggesting that this mutation should affect RNA binding. Most studies of isolated protein domains (vigilin KH6, FMRP KH1, and Nova-2 KH3) have predicted that the I304N mutation results in an unfolded KH domain, which would be expected to lead to loss of specific RNA binding. However, other KH domains harboring mutations analogous to I304N are correctly folded, including the Drosophila homolog of FMRP (dfmr1p) tandem KH1-KH2 [80], and BBP/SF1, in which RNA binding is specifically lost [81]. The first co-crystal of a KH domain—RNA complex (Nova-RNA) [50], as well as a subsequent review of structures [49], suggest that mutation of the conserved hydrophobic amino acid analogous to Ile-304 must decrease RNA binding affinity. While a consensus from these KH domain∶RNA structures is that the isoleucine mutation disrupts KH∶RNA interactions, FMRP has multiple RNA binding domains, so that RNA binding by the full-length protein may not be abrogated despite loss of KH-dependent interactions. This is consistent with our observation that I304N-FMRP in mouse brain fails to crosslink to kcRNA, but retains other activities, including the ability to crosslink to G-quartet RNA and to heterodimerize with FXR1P and FXR2P (Figure 7). While we show that the RGG box in the I304N-FMRP is still competent to bind its high affinity *in vitro*-selected (G-quartet) RNA ligand, it appears from the Superose 6 analysis that I304N-FMRP has lost most of all of its RNA interactions *in vivo*. We propose that RNA binding by the other RNA-binding domains of FMRP may be hierarchical, such that the KH2 domain must make proper RNA interactions for subsequent G-quadruplex binding by the RGG box to occur. Taken together these observations suggest that the I304N-FMRP mutation leads to a global loss of RNA binding *in vivo*, and suggest that identification of FMRP KH2-RNA targets will be of great interest.

### I304N FMRP does not appear to have a dominant negative effect

The severity of Fragile X symptoms reported in the I304N patient has led to the hypothesis that I304N-FMRP might have a dominant negative effect on its autosomal paralogs, FXR1P and FXR2P, decreasing any functional redundancy present in the absence of FMRP. We do not detect any evidence for this as the expression levels, polyribosomal association and mRNP complex sizes of FXR1P and FXR2P are unchanged in the *Fmr1* ^I304N^ mouse brain relative to wild type littermates. At the same time, we find that mutant protein levels vary with age, such that they are reduced (by two-thirds) in adult mice, but are even more markedly reduced at P14, a time when synaptogenesis is occurring in many areas of the mouse brain, including the forebrain and cerebellum. This finding suggests that loss of FMRP activity, including but not necessarily limited to KH2 RNA binding, may play a critical role in leading to the synaptic defects evident in the mouse, and, presumably, in human patients.

In addition, we find that macroorchidism in *Fmr1* ^I304N^ mice, while pronounced compared with wild type littermates, is no more severe than in *Fmr1* null littermates. mGluR-dependent LTD in *Fmr1* ^I304N^ mice is equivalent to that in *Fmr1* null littermates, but not enhanced. Behavioral assays give little or no indication of a more severe behavioral deficit than the *Fmr1* null mouse. Taken together, these findings suggest that *Fmr1* ^I304N^ mice have an *Fmr1* null-like phenotype, consistent with a loss of function mutation. Supporting this, the analogous I307N mutation in Drosophila *dfmr1* results in a partial loss of function phenotype [77]. We propose that the severe Fragile X symptoms, including IQ below 20, lack of verbal communication, and impressive macroorchidism observed in the I304N patient may be a result of selection bias, in that this patient may have been selected for further gene sequencing precisely due to the severity of his Fragile X phenotype. Because screening for Fragile X Syndrome is currently performed by PCR for the CGG repeat expansion, negative results may be classified as nonsyndromic mental retardation or nonspecific developmental delay, in the absence of characteristic features of Fragile X Syndrome.

Although we cannot exclude that the severity of the I304N patient's symptoms may have contributions from other genetic factors, including exacerbation by his familial liver disease, we note that none of the patient's other 29 relatives affected by liver glycogenosis have mental retardation, or the neurologic and phenotypic defects found in the Fragile X patient. We cannot rule out the possibility that the I304N patient might express elevated I304N FMRP levels such that a dominant negative action exacerbates his symptoms. Nonetheless, based on our finding of decreased I304N FMRP in the mouse model and similar results from the I307N mutation in *dfmr1* [77], we infer that it is most likely that the I304N patient has lower steady state levels of neuronal I304N-FMRP.

### A new mouse model for the Fragile X Syndrome

The *Fmr1* ^I304N^ mouse provides an additional mouse model for the Fragile X Syndrome. The most widely used model for Fragile X Syndrome, the *Fmr1^tm1Cgr^* mouse, is a complete null due to the insertion of the neo cassette in exon 5 of the *Fmr1* gene. By causing loss of FMRP expression, the *Fmr1^tm1Cgr^* mutation largely recapitulates the human Fragile X Syndrome at the protein level. Nonetheless, the CGG repeat expansion, present in most human patients, is not replicated in the *Fmr1^tm1Cgr^* null mouse, and the repeat may contribute in unknown ways to the disorder (soaking up CGG DNA binding proteins, or interfering with expression of transcripts present on the other DNA strand [8],[9]). In addition, the *Fmr1^tm1Cgr^* null mouse still contains a neo-expression cassette, which has been documented in some cases to affect expression of neighboring genes and lead to confounding phenotypes. Thus as a model system, the I304N mouse genocopies the human I304N patient better than the null mouse genocopies the CGG repeat expansion. However, we appreciate that human deletions and the point mutation patient, to the extent that they share all the symptoms of Fragile X Syndrome, argue against the CGG repeat expansion itself playing a significant role in the disease, and that the I304N mutation is limited in clinical significance relative to the CGG expansion.

The I304N mutation causes defective KH2-mediated RNA binding in neurons, and decreased FMRP levels, particularly in younger animals. The loss of KH2 function accounts for the dissociation of the protein from brain polyribosomes. We propose that this leads to a loss of proper translational control of FMRP mRNA targets, which in turn leads to the cognitive and behavioral deficits observed in the Fragile X Syndrome. Our observations underscore the importance of identifying FMRP KH2 RNA ligands in the mouse brain to understand the pathogenesis of the disease. Identification of a reliable and comprehensive set of *in vivo* RNA targets will benefit from use of the *Fmr1* ^I304N^ mouse model, in conjunction with the *Fmr1* null [54], *Fmr1* conditional knockout [82], and *FMR1* YAC transgenic mice [53] for validation and functional studies. Finally, trials of potential clinical treatments can be tested on the *Fmr1* ^I304N^ mice, since they provide an additional animal model in which rescue of phenotype can be measured.

## Materials and Methods

### Generation of targeted *Fmr1* I304N knock-in mutation

A genomic clone encoding the murine *Fmr1* gene was isolated from a BAC library derived from a 129 mouse (ES-129/SvJ BAC library, clone address 217I21, Incyte Genomics). To generate an *Fmr1* KH2 I304N targeting vector, a 7.2 kb BamHI-XhoI 5′ homology arm spanning intron 5 to intron 9 and a 1.9kb XhoI-KpnI fragment spanning intron 9 to intron 11 (including exon 10 where the I304N mutation occurs, and a 3′ homology arm (1.3kb)) were cloned into pBluescriptIISK(+) (Stratagene). PCR mutagenesis of the XhoI-KpnI fragment introduced the I304N mutation in exon 10 to change the sequence CTG(Leu) ATT(Ile) CAA(Gln) to CTT(Leu) AAC(Asn) CAG(Gln)). The two wobble mutations were introduced to create a new HindIII site for genotyping and to facilitate PCR genotyping with a mutant-specific primer. A new XbaI site was also generated in the middle of intron 10 in a region that was not conserved between the mouse and human *FMR1* genes. The loxP-Auto-Cre-Neo^R^-loxP (ACNF) cassette (from Dr. Peter Mombaerts) was inserted in the new XbaI site. Finally, the 5′ homology arm and the XhoI-(I304N)-(ACNF)-KpnI fragment were ligated together into the pBluescript plasmid. The plasmid was linearized with NotI and used to electroporate ES cells at the Transgenic Services Laboratory at The Rockefeller University. Genomic DNA from individual colonies was digested with BamHI and screened by Southern blot analysis. The Southern probe overlapping exon 12, which has no corresponding sequence in the mouse *Fxr1* or *Fxr2* gene [83], distinguished the targeted allele, 2.7 kb, from wild type locus, 9.6 kb. Correctly targeted clones were selected for blastocyst injection and transferred to pseudopregnant females, from which germline chimeras were obtained.

*Fmr1* ^tm(I304N)Drnl^ (*Fmr1* ^I304N^) mice were bred greater than 10 generations into FVB and C57BL/6J backgrounds to generate the congenic strains FVB.*Fmr1* ^I304N^ and B6.*Fmr1* ^I304N^. To generate wild-type (wt) and *Fmr1* ^I304N^ mutant littermates on either background, *Fmr1* ^I304N/+^ heterozygous (het) females were bred with wild-type (wt) males of the same background and male offspring were used for experiments. To generate *Fmr1* ^I304N^ and *Fmr1* null littermates *Fmr1* ^I304N/null^ het females were bred with wt male mice and male offspring used.

### RNA preparation

RNA from mouse tissues or polyribosome fractions was extracted using Trizol or Trizol LS Reagent, respectively, according to manufacturer's instructions (Invitrogen). 10ng of *in vitro* translated luciferase RNA was spiked into each polyribosome fraction as a control for RNA recovery. Chloroform∶isoamyl alcohol (49∶1) was added, samples spun at 15min at 12,000×g, the aqueous phase collected and precipitated with ethanol at −20°C overnight. RNA was pelleted at 20,000×g for 20min at 4°C, washed with 75% ethanol, and dissolved in water. RNA was then RQ1 DNase (Promega) treated at 37°C for 1hr and underwent a second round of phenol-chloroform extraction and ethanol precipitation.

### Northern blots

Northern blots were performed following the NorthernMax-Gly protocol (Ambion). Briefly, 30ug of brain RNA and 10ug of testes RNA were denatured with Glyoxal load dye at 50°C for 30min and then were separated on a 0.8% agarose gel in 1× Gel Prep/Gel Running buffer. RNA was transferred to a GeneScreen Plus hybridization transfer membrane (Perkin Elmer) in 10× SSC. ^32^P-labeled *Fmr1* probe (see below) was hybridized to the membrane at 68°C for 2 hrs in QuikHyb hybridization solution (Stratagene). β-actin probe was hybridized at 42°C for 1hr in ULTRAhyb-Oligo solution (Ambion). Membranes were then washed with 2× SSC, 0.1% SDS and 0.1× SSC, 0.1% SDS at room temperature. Radiolabel was detected and quantified by PhosphorImager (Bio-Rad).

*Fmr1* probe, complementary to the 3′UTR, was synthesized by *in vitro* transcription with P^32^-α-UTP using the MAXIscript kit (Ambion) according to the manufacturer's protocol. Template DNA for transcription was generated using a reverse primer that included the T7 promoter sequence (underlined).

F:5′TCAGCAGTATGTTTCAGTCTTTCGG 3′

R:5′TAATACGACTCACTATAGGGGAGAGTTTTCAAAGTTGAAATTCGTCATCAGG 3′

A 20nt long DNA anti-sense probe against β-actin exon 4 was 5′ end labeled with ^32^P-ATP with T4 polynucleotide kinase (NEB) and used for Northern blots.

### Histopathology and testicular measurement

Histopathology analysis was performed by the Rockefeller University Genetically Engineered Mouse Phenotyping core facility. Four male FVB.*Fmr1* ^I304N^ mice, five male FVB.*Fmr1* null littermates and four FVB wild-type mice were sacrificed at 10 weeks of age and tissues fixed in 10% formalin overnight and embedded in paraffin blocks. Tissue sections were deparaffinized, rehydrated, stained with hematoxylin & eosin, and visualized with a Zeiss Axioplan microscope. For testicular analysis, seminiferous tubule diameters were measured and interstitial cell numbers were counted under randomly selected 20× power fields, blind to genotype. Macroorchidism was assessed by combined weight of both testes of *Fmr1* ^I304N^ mice compared to that of either wt or *Fmr1* null littermates at indicated ages. Measurements from multiple litters of similar ages were pooled and subjected to statistical analysis (student's t-test). Both histology and testicular size measurements were performed using mice bred greater than 10 generations into the C57BL/6 background (B6.*Fmr1* null breeding pairs were generously provided by Dr. W. Greenough, U. Illinois)).

### Behavioral analysis

B6.*Fmr1* ^I304N/+^ heterozygous female mice were shipped to Baylor College of Medicine where they were embryo rederived by mating with male C57BL/6J mice. Female offspring were backcrossed an additional generation to C57BL/6J male mice. Mice used for behavioral analysis were generated by mating these mice with B6.*Fmr1* ^I304N/+^ heterozygous females and were maintained at the same B6 backcross generation. All mice started testing between 2–3 months of age by experimenters blind to the genotype of the mice using behavioral protocols previously described [53],[68]. Twenty-three mutant and 18 wild-type littermates were evaluated for this study.

Behavioral results were analyzed using SPSS. Data were analyzed using one- and two-way ANOVA with repeated measures as appropriate. Significant interactions were analyzed using simple-effects follow-up comparisons. Levels of significance were set at p≤0.05.

### Electrophysiology

Hippocampal slices (400µm) were prepared from 30–90 day old B6.*Fmr1* ^I304N^ mice and their wt or B6.*Fmr1* null littermates as described [11],[61]. All experiments were performed blind to genotype and in the presence of the NMDA receptor antagonist D,L-AP5 (50 µM; Tocris) to isolate mGluR-dependent LTD. D,L-AP5 and anisomycin were prepared fresh daily in artificial cerebrospinal fluid which consists of (in mM) NaCl, 124; KCl, 5; NaH_2_PO_4_, 1.25; NaHCO_3_, 26; MgCl_2_, 1; CaCl_2_, 2; dextrose, 10. Extracellular field potentials (FPs) were measured in the stratum radiatum of hippocampal CA1 elicited by Schaffer collateral stimulation. mGluR-LTD was induced by application of 100µM DHPG for 5min or by pairs of stimuli (50 msec interstimulus interval) delivered at 1 Hz for 20 min (2400 pulses; PP-LFS). Synaptic strength was measured as the initial slope (10–40% of the rising phase) of the FP. LTD magnitude was compared at 60–70min after the onset of DHPG or PP-LFS. Slices were preincubated in antagonists or inhibitors for 20–30 min before DHPG or PP-LFS. The effects of all pharmacological treatments on LTD were evaluated by comparing interleaved control and treated slices. Independent t-tests were used to determine statistical significance.

### Total protein extract for western blot

*Fmr1* ^I304N^ mice and their wild type littermates on the FVB background were used for determining FMRP levels by western blot. Tissues were Dounce homogenized in lysis buffer (0.5% NP-40, 0.5% deoxycholic acid, 0.1% SDS in PBS, 50% glycerol, and 1× Complete protease inhibitor cocktail (Roche)) sonicated to fully disrupt nuclei, shear nucleic acids and disrupt macromolecular complexes, and spun at 20,000×g at 4°C for 15min. Protein concentration was determined by Bradford assay compared with BSA standards. 50ug of protein from each sample was boiled in SDS-sample buffer and used for western analysis.

### Antibodies

The following antibodies were used throughout the work: anti-FMRP mab2160 at 1∶1000 for immunoblot (IB) (Chemicon), anti-FMRP 2F5 at 1∶100 for IB [84], anti-FMRP ab17722 at 1∶1000 for immunoblot (Abcam), anti-FMRP 7G1-1 ascites (Developmental Studies Hybridoma Bank (DSHB), University of Iowa) at 1∶100 for IB, also used for IP as described [85],[86], anti-FXR1P ML13 at 1∶10,000 for IB (gift from Dr. E. Khandjian (Université Laval, Quebec)), anti-FXR2P 1G2 concentrated supernatant at 1∶100 for IB (DSHB), anti-hsp90 at 1∶5000 for IB (BD Biosciences), anti-gamma tubulin (GTU-88, Sigma) at 1∶10,000 for IB, anti-ribosomal S6 protein at 1∶1000 for IB (Cell Signaling), human patient serum against Nova at 1∶1000 for IB [87], HRP-conjugated anti-His-tag at 1∶5000 for IB (Novagen). HRP-conjugated anti-mouse, anti-rabbit, or anti-human IgG at 1∶10,000 for IB (Jackson Immunoresearch).

### Western blot

Samples were run on 8% or 4–12% gradient SDS-polyacrylamide gels and transferred to Immobilon-P membranes (Millipore) by standard methods. Membranes were blocked for 1hr at room temperature in 10% non-fat dry milk in western blot wash buffer (WBWB) (23mM Tris, pH 8.0, 190mM NaCl, 0.1% w/v BSA, 1mM EDTA, 0.5% Triton X-100, 0.02% SDS). Primary antibodies in 10% milk in WBWB were used during incubation for 1 hr at room temperature or overnight at 4°C. Blots were washed with WBWB 5 times for 5 min after each antibody incubation. Signals were detected by enhanced chemiluminescence (Western Lightning detection kit, Perkin Elmer) and quantified with a Versadoc Imaging System (Bio-Rad).

### Quantitative RT–PCR

Purified RNA was reverse transcribed using random hexamers (Roche) and Superscript III reverse transcriptase (Invitrogen) according to manufacturer's protocols. cDNA products were amplified using SYBR green PCR master mix (Applied Biosystems) with 200nM of the following primers.

*Fmr1* 1F (spanning exon 2 to 3) 5′-TGAAAACAACTGGCAACCAGAGAG-3′

*Fmr1* 1R (spanning exon 2 to 3) 5′-CAGGTGGTGGGAATCTCACATC-3′

*Fmr1* 2F (spanning exon 10 to 11) 5′-GTCAGGAGTTGTGAGGGTGAGG-3′

*Fmr1* 2R (spanning exon 10 to 11) 5′-GGAAGGTAGGGAACTTGGTGGC-3′

*Gapdh* F 5′-CATGGCCTTCCGTGTTCCTA-3′

*Gapdh* R 5′-GCGGCACGTCAGATCCA-3′

*Luc* F 5′-GCCTTGATTGACAAGGATGGA-3′

*Luc* R 5′-CAGAGACTTCAGGCGGTCAAC-3′

Quantitative PCR amplification was performed using a 7900HT sequence detection system (Applied Biosystems) at the Genomic Resource Center at The Rockefeller University. Fluorimetric intensity of SYBR green was monitored during each cycle of amplification to quantify mRNA levels. Regression curves were drawn for each sample and relative amount of mRNA was calculated from the threshold cycles using the instrument's software, SDS 2.0. For expression levels in total brain, relative levels of *Fmr1* mRNA were measured using the standard curve method and normalized to the internal control GAPDH mRNA. Three pairs of littermates were used for *Fmr1* mRNA determination by Q-PCR. Error was calculated using the formula suggested by the ABI user bulletin, [(std dev for *Fmr1*)^2^ + (std dev for *Gapdh*)^2^]^0.5^ For polyribosome distribution, relative *Fmr1* or *Gapdh* mRNA level in each fraction was normalized to spiked-in luciferase RNA analyzed using the ΔΔCt method. The amount of *Fmr1* mRNA in each fraction was then plotted as a percentage of total *Fmr1* mRNA summed over the entire polyribosome gradient. Q-PCR experiments were each repeated 2 times with two pairs of biologic replicates. Error bars reflect the technical replicates from triplicate wells in a single representative experiment from polysome gradients. Error is calculated using the formula suggested by the ABI user bulletin, [(std dev for *Fmr1*)^2^ + (std dev for luc)^2^]^0.5^. Because of gradient variability from day to day they cannot be plotted together so a representative experiment is shown.

### Mouse brain polyribosome analysis

Mouse brain polyribosomes were prepared essentially according to established protocols [27],[51]. Briefly, 2 week-old mice were sacrificed by isoflurane anaesthesia and decapitation. The brain was removed and placed in ice-cold dissection buffer (10mM HEPES-KOH, pH 7.4, 150mM KCl, 5mM MgCl_2_, 100ug/ml cycloheximide). Cortex and cerebellum were dissected free of underlying white matter, homogenized in 1ml lysis buffer (10mM HEPES-KOH, pH 7.4, 150mM KCl, 5mM MgCl_2_, 0.5mM dithiothreitol, 100ug/ml cycloheximide, 1× Complete EDTA-free protease inhibitor cocktail (Roche), 40U/ml rRNAsin (Promega)) per brain with 12 strokes at 900rpm in a motor-driven Teflon-glass homogenizer. The homogenate was spun at 2000×g for 10min at 4°C. The supernatant (S1) from the homogenized material was collected and adjusted to 1% NP-40, incubated for 5min on ice, and spun at 20,000×g for 10min at 4°C. The resulting supernatant (S2) was loaded onto a 20–50% w/w linear density gradient of sucrose in 10mM HEPES-KOH pH 7.4, 150mM KCl, and 5mM MgCl_2_. Gradients were centrifuged at 40,000 rpm for 2 hrs at 4°C in a Beckman SW41 rotor. Fractions of 0.75ml volume were collected with continuous monitoring at 260nm using an ISCO UA-6 UV detector. 400 ul of each fraction was TCA precipitated and analyzed by Western blot.

### Superose 6 gel filtration

A pre-packed Superose 6 Precision column PC 3.2/30 in a SMART system (GE Healthcare) was used to determine the molecular masses of protein complexes. The optimal separation range of globular proteins in this column is 5 kDa to 5000 kDa with an exclusion limit of 40,000 kDa. Mouse brain cytoplasmic lysates (0.3% NP-40, 10mM HEPES, pH 7.4, 150mM KCl, either with or without 30mM EDTA) were spun over a 0.22um Spin-X column (Corning) before loading onto a Superose 6 column with a flow rate of 30ul/min. Protein profile was monitored at A280nm and fractions of 75ul were collected. Fractions were TCA precipitated for Western analysis. To calibrate the column, protein markers (GE Healthcare) were run and gave the following results: blue dextran (void ≥40,000 kDa) in fraction 4, 669 kDa at the fraction 10/11 boundary, 440 kDa in fraction 13, 67 kDa in fraction 15/16 and 13.7 kDa in fraction 18. Molecular mass was extrapolated by linear regression analysis for each fraction according to the migration of these protein markers and used for identification of protein complex sizes we studied. The average MW in each fraction was: fraction 12 (463 kDa), 13 (261 kDa), 14 (148 kDa), 15 (83 kDa) and 16 (47 kDa). For complete RNase digestion, recombinant RNase A and T1 (Ambion) were added to mouse brain lysates to a final concentration of 20 ug/ml and 10,000 U/ml respectively. Lysates were incubated at room temperature for 30 min before gel filtration.

### Co-immunoprecipitation of FMRP and FXR1/2P

A 1∶1 slurry of protein A Sepharose beads (Sigma) was first bound to 120 ug of rabbit anti-mouse Fcγ bridging antibody (Jackson ImmunoResearch) for 20 minutes at room temperature, washed 3 times in 0.1 M phosphate buffer pH 8.1, and bound to 10 ul of anti-FMRP monoclonal 7G1-1 ascites (5mg/ml, DSHB, U. Iowa). Brain cytoplasmic lysates were prepared in lysis buffer (0.5% Triton X-100, 30mM HEPES, pH 7.4, 200mM NaCl, 30mM EDTA, and Complete protease inhibitor cocktail (Roche)), by Dounce homogenization of one adult brain in 2 ml lysis buffer and centrifugation at 20,000×g for 10 minutes at 4 degrees, and were incubated with beads and antibodies at 4°C for 2 hrs. Immunoprecipitates were washed four times with the same lysis buffer. Sepharose beads were heated to 95° in SDS sample loading buffer and supernatants analyzed by Western analysis.

### T7 transcription and directed UV crosslinking IP

96nt long G-quartet (GQ) and kissing complex (kc) RNAs [51],[64] were *in vitro* transcribed with P^32^-α-UTP and P^32^-α-GTP. RNAs were gel purified on denaturing gels and spiked into 100ul S2 brain lysate prepared as for polyribosome analysis from 2 month old mice of the indicated genotype. RNAs were incubated with the brain lysates for 15 min at room temperature and then UV crosslinked as described [88]. Lysates were then diluted with 500 ul stringent IP buffer (0.5% NP-40, 0.5% deoxycholic acid, 0.1% SDS in PBS) and the IP was performed as for the co-immunoprecipitation, with the exception that stringent IP buffer was both the IP and wash buffer. Immunoprecipitates were run on 4–12% SDS-PAGE, transferred to nitrocellulose membranes, and exposed by autoradiography.

## Supporting Information

Figure S1 Light micrographs of sagittal sections of Fmr1^I304N^ knock-in mouse brains and testes reveal no microscopic abnormality. Post mortem mouse organs were fixed in 10% formalin, paraffin embedded, sectioned, and stained with hematoxylin & eosin. (A,B) Wild-type cerebellum, (C,D) I304N cerebellum, (E,F) wild-type cortex, (G,H) I304N cortex, (I,J) wild-type hippocampus, (K,L) I304N hippocampus, (M,N) wild-type testes, and (O,P) I304N testes and photographed at two magnifications. FVB. Fmr1^I304N^ mice have normal seminiferous tubular diameter (wild type tubular diameter = 177.5±21.4 µm, n = 30 and I304N tubular diameter = 176.0±17.3 µm, n = 30, p>0.05), normal interstitial mass without edema (wild-type interstitial cell number = 33±8, n = 10, I304N interstitial cell number = 32±5, n = 10, under 20× field, p>0.05), and normal spermatogenesis.
(0.85 MB PDF)

Figure S2 Behavorial assays in Fmr1^I304N^ mice and their wild type littermates. The findings for both day 1 and day 2 were similar; day 1 data are presented, and details for each assay are given below. Assays performed were as follows. Activity in open field test: (A) total distance traveled, (B) vertical distance (rearing). Anxiety related responses: (C) center∶total distance ratio in an open field, (D) light to dark transition, (E) time spent in the dark chamber. Startle habituation: (F) acoustic startle response in PPI test, (G) %PPI with increasing prepulse level. Conditioned fear: (H) number of freezing bouts in the context test, (I) number of freezing bouts in the acoustic conditioned stimulus test. (J) Hotplate test for sensitivity to pain as measured by latency of response. (K) Number of marbles buried as a measure of obsessive-compulsive behavior. (L) % of mice displaying audiogenic seizure in response to a stimulus.
(0.07 MB PDF)

Text S1 Locomotor activity in an open field, anxiety related responses, acoustic startle and prepulse inhibition of the startle, conditioned fear, hotplate, marble bury, audiogenic seizure.
(0.04 MB DOC)
