# Supplementary figures and images for: A Mouse Model of the Human Fragile X Syndrome I304N Mutation
Source: PLoS Genet. 2009 Dec 11;5(12):e1000758. doi: 10.1371/journal.pgen.1000758 (PMC2779495; doi:10.1371/journal.pgen.1000758)

Supplementary Figure S1.

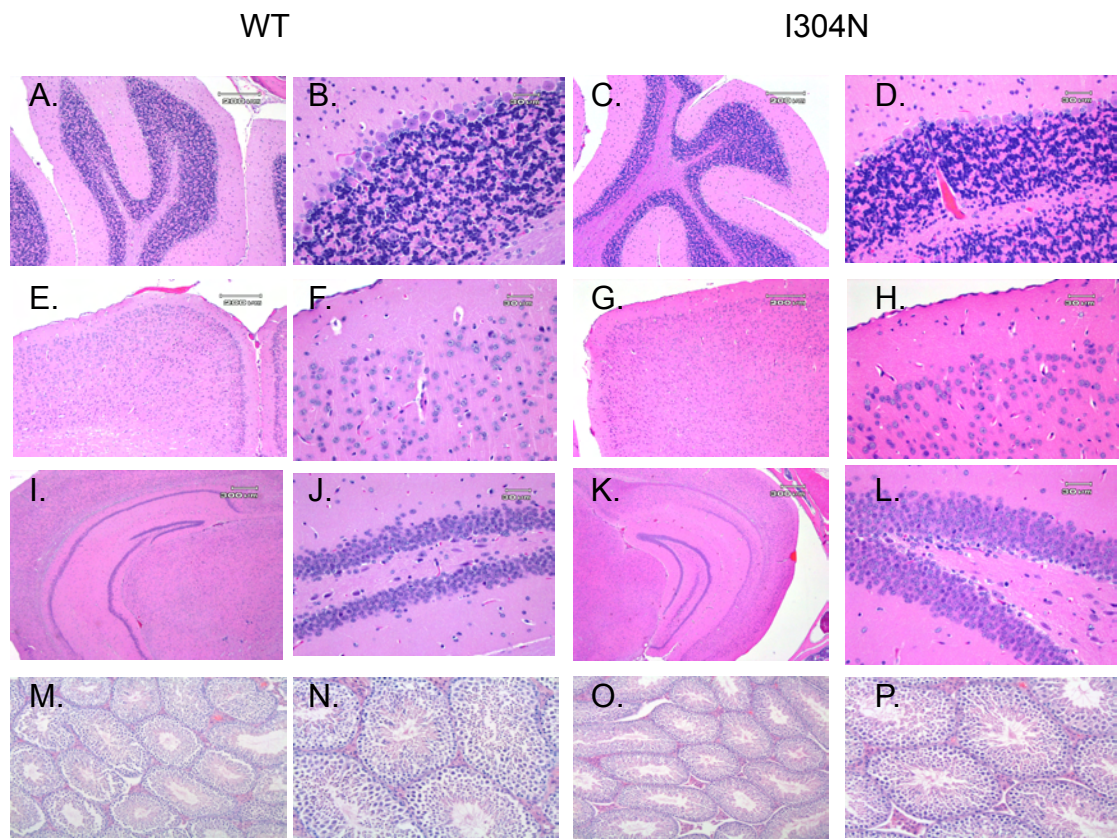

Supplement: Figure S1 — Light micrographs of sagittal sections of Fmr1I304N knock-in mouse brains and testes reveal no microscopic abnormality. Post mortem mouse organs were fixed in 10% formalin, paraffin embedded, sectioned, and stained with hematoxylin & eosin. (A,B) Wild-type cerebellum, (C,D) I304N cerebellum, (E,F) wild-type cortex, (G,H) I304N cortex, (I,J) wild-type hippocampus, (K,L) I304N hippocampus, (M,N) wild-type testes, and (O,P) I304N testes and photographed at two magnifications. FVB. Fmr1I304N mice have normal seminiferous tubular diameter (wild type tubular diameter = 177.5±21.4 µm, n = 30 and I304N tubular diameter = 176.0±17.3 µm, n = 30, p>0.05), normal interstitial mass without edema (wild-type interstitial cell number = 33±8, n = 10, I304N interstitial cell number = 32±5, n = 10, under 20× field, p>0.05), and normal spermatogenesis. (0.85 MB PDF) [file pgen.1000758.s001.pdf]

Supplementary Figure S2.

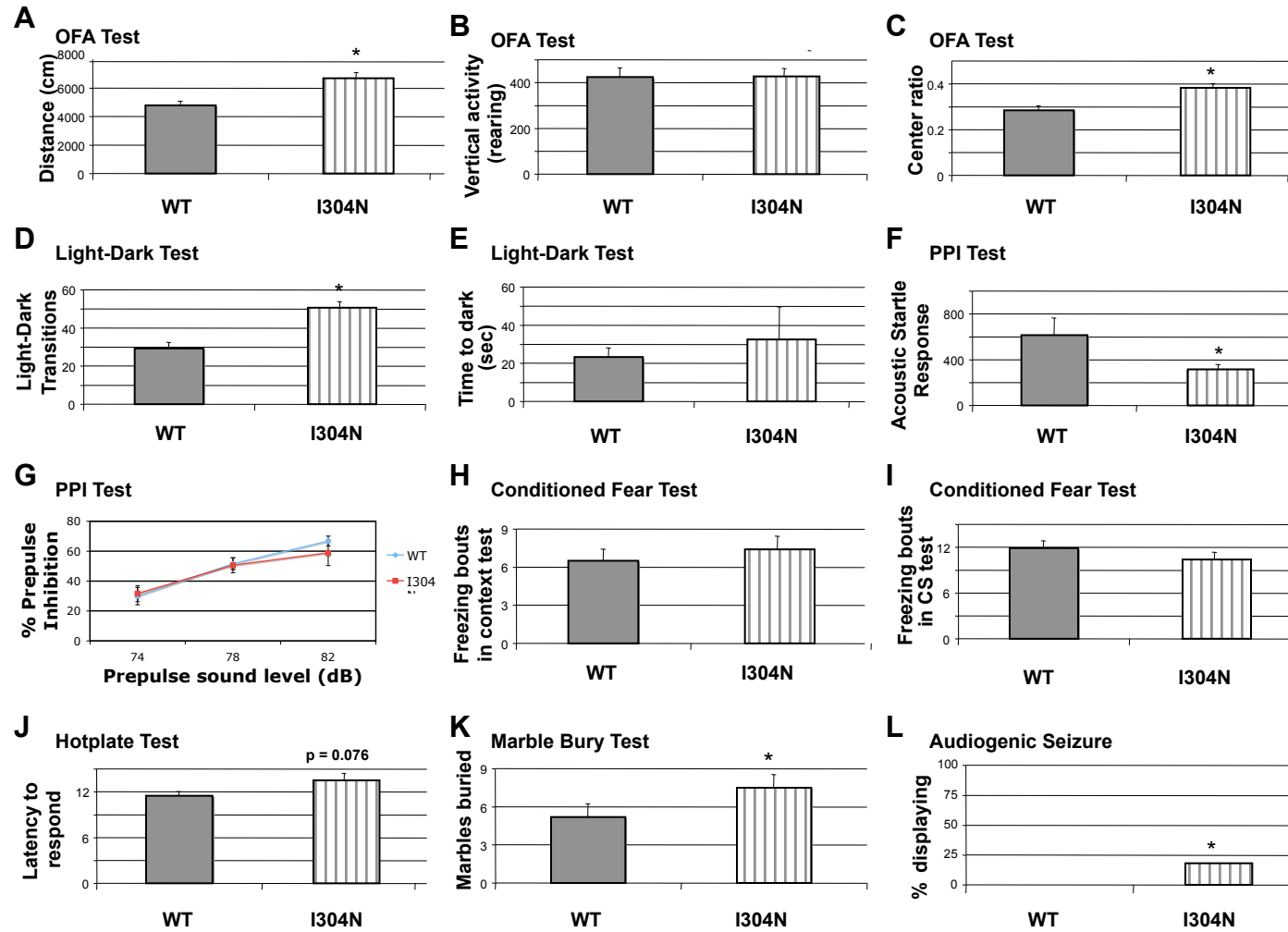

Supplement: Figure S2 — Behavorial assays in Fmr1I304N mice and their wild type littermates. The findings for both day 1 and day 2 were similar; day 1 data are presented, and details for each assay are given below. Assays performed were as follows. Activity in open field test: (A) total distance traveled, (B) vertical distance (rearing). Anxiety related responses: (C) center∶total distance ratio in an open field, (D) light to dark transition, (E) time spent in the dark chamber. Startle habituation: (F) acoustic startle response in PPI test, (G) %PPI with increasing prepulse level. Conditioned fear: (H) number of freezing bouts in the context test, (I) number of freezing bouts in the acoustic conditioned stimulus test. (J) Hotplate test for sensitivity to pain as measured by latency of response. (K) Number of marbles buried as a measure of obsessive-compulsive behavior. (L) % of mice displaying audiogenic seizure in response to a stimulus. (0.07 MB PDF) [file pgen.1000758.s002.pdf]
