# Supplementary material for: A Mouse Model of the Human Fragile X Syndrome I304N Mutation
Source: PLoS Genet. 2009 Dec 11;5(12):e1000758. doi: 10.1371/journal.pgen.1000758 (PMC2779495; doi:10.1371/journal.pgen.1000758)
Supplement: Text S1 — Locomotor activity in an open field, anxiety related responses, acoustic startle and prepulse inhibition of the startle, conditioned fear, hotplate, marble bury, audiogenic seizure. (0.04 MB DOC) [file pgen.1000758.s003.doc]

**Supplementary Text S1**.

*Locomotor activity in an open field.* Open-field tests assess exploratory behavior in a novel environment. Mice were placed in the open-field arena and their activity recorded over a period of 30 min on two consecutive days. *Fmr1^I304N^* mice traveled a greater total distance in the open field compared with their wild type littermates (p=0.001) (**A**) suggesting that they had increased exploratory behavior and locomotor activity. There was no difference between *Fmr1^I304N^* and wild type mice for rearing, as measured by vertical activity (p=0.927) (**B**).

*Anxiety related responses.* The center:total distance ratio in the open field test is a measurement of anxiety-related response to a brightly-lit open arena. Wild type mice spent a greater proportion of their total distance traveling along the perimeter of the arena while *Fmr1^I304N^* mice traveled a greater proportion in the center of the open field (p < 0.001) indicating a decrease in anxiety relative to normal mice (**C**). *Fmr1* null mice also spend a greater proportion of their total distance traveled away from the walls of the arena [1,2]. In the light and dark exploration test, another measure of anxiety response, mice were allowed to move freely between a large, open, brightly lit chamber and a small, closed dark chamber. *Fmr1^I304N^* mice exhibited a greater number of light-dark transitions than their wild type littermates (p < 0.001) (**D**). No significant difference was observed between *Fmr1^I304N^* and wild type littermates for the total time spent in the dark (p > 0.05) (**E**). Taken together, *Fmr1^I304N^* mice had a lower level of anxiety than WT littermates, consistent with previous results in *Fmr1* null mice [1-3].

*Acoustic startle and prepulse inhibition of the startle.* Prepulse inhibition (PPI) of the acoustic response was used to measure sensorimotor gating. A weak non-startling sound presented immediately before a startling sound suppresses the startle response in wild type mice, and this suppression is diminished in *Fmr1* null mice [1,2,4-7]. *Fmr1^I304N^* mice exhibited significantly lower startle compared to their wild type littermates (p= 0.031; **F**), consistent with results in *Fmr1* null mice, but had no significant difference in PPI responses (p=0.701; **G**).

*Conditioned fear.* The conditioned fear test, based on a Pavlovian learning and memory paradigm, was used to examine cognitive functions. In this test mice are placed into a test chamber and after two minutes presented two auditory conditioned stimuli (CS) followed by a mild footshock, the unconditioned stimulus (US). Twenty four hours later mice are placed back into the same test chamber and monitored for conditioned freezing in the test context (context test). One hour later mice are placed back into a novel chamber, and given the auditory cue (CS) and their freezing bouts measured (CS test). *Fmr1^I304N^* mice displayed no difference in conditioned fear in either the context or the auditory CS tests compared with wild type littermates (p > 0.05; **H-I**), consistent with previous results in *Fmr1* null mice from our laboratory [4].

*Hotplate.* The hotplate test was used to assess pain sensitivity. Mice were placed on a 55^o^C hotplate and the latency of their first hindlimb response was recorded. *Fmr1^I304N^* mice showed a greater latency to hindlimb response than wild type mice, however, this effect failed to reach statistical significance (p=0.076; **J**), suggesting they may be less sensitive to pain. Previous studies have also found a slightly greater latency to hindlimb response in the *Fmr1* null mouse that was not statistically significant [4].

*Marble bury.* Assays of marble burying test for repetitive/perseverative responses in the presence of novel or familiar objects. It is important to note that marble burying is not simply another anxiety test since it is not genetically correlated with either open-field or light-dark box exploration (R. Paylor, unpublished observations); such perseveration may instead relate to frontal cortex dysfunction. *Fmr1^I304N^* mice buried significantly more marbles than their wild type littermates (p = 0.046) (**K**), indicating an increase in repetitive/perseverative responses, consistent with observations in *Fmr1* null mice (Paylor, unpublished observations).

*Audiogenic seizure.* Increased audiogenic seizure susceptibility is one of the most robust phenotypes in Fragile X null mice and may be related to the increased cortical excitability seen in the human disease. Approximately 20-25% of Fragile X patients have benign focal epilepsy of childhood [8,9]. Audiogenic seizures have been documented in *Fmr1* null animals on FVB/N, C57Bl/6J and F1 crossed genetic backgrounds and from several independent laboratories [10-14]. While *Fmr1* KO mice on the FVB background show a high incidence of audiogenic seizures well into adulthood, *Fmr1* KO mice on a C57BL/6J genetic background do not display seizures after 7 weeks of age [14]. We found that 18% of our 2-3 month old *Fmr1^I304N^* mice had audiogenic seizures, while none were observed in wild-type littermates (**L**). The presence of seizures in the adult *Fmr1^I304N^* mice suggests that they may have more severe cortical excitability than *Fmr1* KO mice on a comparable genetic background, although the relatively low incidence may reflect the tail end of seizure susceptibility period in this background.

**Supplementary References:**

1. Peier AM, McIlwain KL, Kenneson A, Warren ST, Paylor R et al. (2000) (Over)correction of FMR1 deficiency with YAC transgenics: behavioral and physical features. Hum Mol Genet 9: 1145-1159.

2. Qin M, Kang J, Smith CB (2005) A null mutation for Fmr1 in female mice: effects on regional cerebral metabolic rate for glucose and relationship to behavior. Neuroscience 135: 999-1009.

3. Mineur YS, Sluyter F, de Wit S, Oostra BA, Crusio WE (2002) Behavioral and neuroanatomical characterization of the Fmr1 knockout mouse. Hippocampus 12: 39-46.

4. Spencer CM, Serysheva E, Yuva-Paylor LA, Oostra BA, Nelson DL et al. (2006) Exaggerated behavioral phenotypes in Fmr1/Fxr2 double knockout mice reveal a functional genetic interaction between Fragile X-related proteins. Hum Mol Genet 15: 1984-1994.

5. Yun SW, Platholi J, Flaherty MS, Fu W, Kottmann AH et al. (2006) Fmrp is required for the establishment of the startle response during the critical period of auditory development. Brain Res 1110: 159-165.

6. Frankland PW, Wang Y, Rosner B, Shimizu T, Balleine BW et al. (2004) Sensorimotor gating abnormalities in young males with fragile X syndrome and Fmr1-knockout mice. Mol Psychiatry 9: 417-425.

7. Paylor R, Yuva-Paylor LA, Nelson DL, Spencer CM (2008) Reversal of sensorimotor abnormalities in FMR1 knockout mice carrying a human FMRP transgene. Behav Neurosci in press:

8. Berry-Kravis E (2002) Epilepsy in fragile X syndrome. Dev Med Child Neurol 44: 724-728.

9. Musumeci SA, Hagerman RJ, Ferri R, Bosco P, Dalla Bernardina B et al. (1999) Epilepsy and EEG findings in males with fragile X syndrome. Epilepsia 40: 1092-1099.

10. Dolen G, Osterweil E, Rao BS, Smith GB, Auerbach BD et al. (2007) Correction of fragile X syndrome in mice. Neuron 56: 955-962.

11. Musumeci SA, Bosco P, Calabrese G, Bakker C, De Sarro GB et al. (2000) Audiogenic seizures susceptibility in transgenic mice with fragile X syndrome. Epilepsia 41: 19-23.

12. Chen L, Toth M (2001) Fragile X mice develop sensory hyperreactivity to auditory stimuli. Neuroscience 103: 1043-1050.

13. Yan QJ, Asafo-Adjei PK, Arnold HM, Brown RE, Bauchwitz RP (2004) A phenotypic and molecular characterization of the fmr1-tm1Cgr fragile X mouse. Genes Brain Behav 3: 337-359.

14. Yan QJ, Rammal M, Tranfaglia M, Bauchwitz RP (2005) Suppression of two major Fragile X Syndrome mouse model phenotypes by the mGluR5 antagonist MPEP. Neuropharmacology 49: 1053-1066.
